# ART-DEX: A novel strategy to monitor broadly neutralizing antibody activity during antiretroviral therapy of HIV-1

**DOI:** 10.1016/j.xpro.2024.103056

**Published:** 2024-08-30

**Authors:** Magdalena Schwarzmüller, Alexandra Trkola

**Affiliations:** 1 Institute of Medical Virology, University of Zurich, Zurich, Switzerland

**Keywords:** Cell Biology, Cell culture, High Throughput Screening, Immunology, Microbiology, Antibody

## Abstract

Therapeutic use of HIV-1 broadly neutralizing antibodies (bnAbs), passively administered or induced by therapeutic vaccines, is a focus of advanced treatment strategies under development. To enable monitoring of bnAb activity during concurrent antiretroviral therapy (ART), we developed ART-DEX, an analytic strategy that allows high-throughput detection of pure antibody-based neutralizing activity. ART-DEX combines pH-dependent dissociation of antiretrovirals (ARVs) from plasma proteins and size exclusion to effectively remove ARVs from plasma samples, reducing the confounding effects of ARVs on neutralization assays.

For complete details on the use and execution of this protocol, please refer to Schwarzmüller et al.^1^

## Highlights

• ART-DEX releases ARVs from plasma proteins to remove them from plasma samples
• ART-DEX reduces the confounding effects of ARVs on neutralization assays
• ART-DEX allows high-throughput analysis of plasma neutralization of ART-treated PWH

## Abstract (editor highlights)

Publisher’s note: Undertaking any experimental protocol requires adherence to local institutional guidelines for laboratory safety and ethics.

## Graphical abstract

**Figure:**
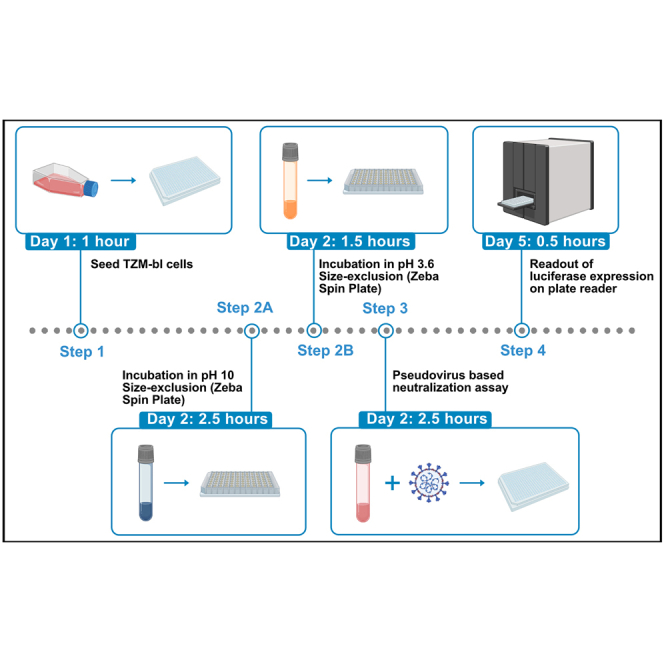

## Before you begin

### Familiarize yourself with the standard TZM-bl HIV-1 neutralization protocol

The ART-DEX method described here builds on the standard protocols for TZM-bl neutralization assays with HIV-1 pseudoviruses widely used in the HIV-1 field.^2^^,^^3^ TZM-bl cells are HeLa cells genetically engineered to stably express CD4 and CCR5 next to endogenous CXCR4. They also contain a Tat-inducible HIV long terminal repeat (LTR)-luciferase reporter gene that is activated upon HIV infection. Neutralization assays performed with TZM-bl cells have been highly standardized and validated.^2^^,^^3^ Take particular note of the biosafety and ethical requirements when working with plasma from people with HIV (PWH).

### Culture of TZM-bl and HEK 293-T cells

**Timing: 2 weeks**

1. Thaw TZM-bl and HEK 293-T cells.
2. Culture cells in Dulbecco’s Modified Eagle Medium (DMEM) containing 10% FCS, 100 U/mL penicillin, and 100 μg/mL streptomycin (DMEM-10).
3. Remove media from 75 cm^2^ cell culture flask.
4. Wash cells with 10 mL DPBS.
5. Add 2 mL Trypsin-EDTA and incubate at 37°C until cells are detached (around 5 min for TZM-bl cells and 1–2 min for HEK 293-T cells).
6. Resuspend detached cells in 13 mL DMEM-10.
7. Centrifuge cells for 10 min at 300 × *g*.
8. Resuspend cell pellet in 10 mL fresh DMEM-10.
9. Transfer the required dilution (1:5 for TZM-bl and 1:20 HEK 293-T cells for splitting every 3–4 days) of the cell suspension into a new 75 cm^2^ cell culture flask.

***Note:*** We typically freeze TZM-bl and HEK 293-T cells at densities of 2–3 × 10^6^. Cells in culture are passaged 2–3 times per week. We recommend a maximum passage number of 20 and 40 for TZM-bl and HEK 293-T cells, respectively.

***Note:*** Cells have to be tested regularly for mycoplasma contamination.

### Preparation of pseudoviruses

**Timing: 1 week**

10. Seed 2.25 × 10^6^ HEK 293-T cells per 75 cm^2^ cell culture flask.
11. Incubate cells overnight at 37°C, 5% CO_2_.
12. Transfect HEK 293-T cells.
  a. Mix 5 μg plasmid encoding for Env protein and 15 μg pseudovirus backbone vector in 4 mL DMEM.
  b. Add 60 μg polyethyleneimine MAX.
  c. Incubate transfection mix for 20 min at room temperature.
  d. Carefully add transfection mix to HEK 293-T cells.
  e. Incubate cells for 8 h at 37°C, 5% CO_2_.
  f. Add fresh DMEM-10 to cells.
  g. Incubate cells for 48 h at 37°C, 5% CO_2_.

***Note:*** Pseudovirus backbone NLluc-AM^4^ and the multidrug-resistant version of NLluc-AM (HIV^pv^-MDR, Schwarzmüller et al.^1^) have been pseudotyped with diverse Env (Murine Leukemia Virus (MuLV) and HIV) and probed in combination with ART-DEX.

***Note:*** It is highly recommended to include pseudoviruses carrying an Env of an unrelated virus (e.g., MuLV) as control.

13. Harvest pseudovirus.
  a. Sterile filtrate cell culture supernatant containing pseudoviruses using Steriflip filter units (0.22 μm).
  b. Store pseudovirus in aliquots at −80°C.
14. Determine infectivity of frozen pseudovirus stocks by serial dilution.
  a. Serially dilute frozen pseudovirus stocks in DMEM-10 starting with undiluted pseudovirus (e.g., 1:4 dilution for 5 dilution steps).
  b. Add 100 μL of virus dilution to 100 μL TZM-bl cells seeded in 96 well plates (1 × 10^4^ cells/well, see steps 1-3, seeding TZM-bl cells).
  c. Incubate for 48–72 h at 37°C, 5% CO_2_.
  d. Carefully remove media from cells.
  e. Add 50 μL Luciferase lysis buffer to each well and incubate for 5 min.
  f. Add 50 μL Bright-Glo luciferase assay substrate (diluted 1:10 in Luciferase lysis buffer) per well.
  g. Measure luciferase expression on the PerkinElmer plate reader within 30 min.
  h. To calculate infectivity of pseudovirus stocks, normalize background-corrected RLU values to virus input and calculate average titer (RLU/μL) over the linear range of the dilution curve.

***Note:*** Minimal titers should be above 100 RLU/μL, optimal titers are above 500 RLU/μL.

**Pause point:** Pseudovirus stocks can be stored at −80°C until use in neutralization assays.

### Heat-inactivation of plasma samples

**Timing: 1 h**

15. All plasma or serum samples irrespective if from people with or without HIV must be incubated for 1 h in a water bath at 56°C to inactivate HIV, potentially other pathogens, complement and coagulation factors.

***Note:*** Prior to use in neutralization assay, store plasma sample for at least 2 h at −80°C.

**Pause point:** Heat-inactivated plasma can be stored at −80°C until use in neutralization assays.

## Key resources table

**Table:** 

REAGENT or RESOURCE	SOURCE	IDENTIFIER
**Bacterial and virus strains**		

HIV-1 Env plasmid of choice (e.g., Panel of Global HIV-1 Env clones)	NIH AIDS Reagent Program	HRP-12670
Non-HIV plasmid of choice (e.g., MuLV)	NIH AIDS Reagent Program	ARP-1065
HIV pseudotyping backbone of choice (e.g., NLluc-AM)	Provided by A. Marozsan and J.P. Moore (Pugach et al.^4^)	N/A
Multidrug-resistant HIV pseudotyping backbone of choice (e.g., HIV^pv^-MDR)	Schwarzmüller et al.^1^	N/A

**Biological samples**		

Human plasma or serum samples	N/A	N/A

**Chemicals, peptides, and recombinant proteins**		

DMEM, high glucose, pyruvate	Thermo Fisher Scientific	41966052
Trypsin-EDTA (0.25%)	Thermo Fisher Scientific	25200056
HEPES (1 M)	Thermo Fisher Scientific	15630056
DPBS	Thermo Fisher Scientific	14190094
Polyethyleneimine MAX	Polysciences	24765-1
Diethylaminoethyl-dextran hydrochloride	Merck KGaA	D9885

**Critical commercial assays**		

Zeba Spin desalting plates, 40K MWCO	Thermo Fisher Scientific	A57767
Bright-Glo Luciferase assay system	Promega	E2650

**Experimental models: Cell lines**		

TZM-bl cells	NIH AIDS Reagent Program	ARP-8129
HEK 293-T cells	ATCC	CRL-3216

**Software and algorithms**		

GraphPad Prism 10.1.0	GraphPad Software	N/A

**Other**		

Steriflip filter unit (0.22 μm)	Merck KGaA	SCGP00525
384 white well, optically clear culture plates	Greiner Bio-One	781098
EnVision 2104 multilabel reader	PerkinElmer	N/A

## Materials and equipment

**Table:** Phosphate buffer, pH 10.0

Reagent	Amount
Sodium Phosphate Dibasic Heptahydrate	2.021 g
Sodium Phosphate Monobasic Monohydrate	339 mg
NaOH	Adjust to pH 10.0
ddH_2_O	Up to 100 mL

Store at room temperature for up to 1 month.

**Table:** Acetate buffer, pH 3.6

Reagent	Amount
Sodium Acetate	39 mg
Acetic Acid	572 mg
HCl	Adjust to pH 3.6
ddH_2_O	Up to 100 mL

Store at room temperature for up to 1 month.

**Table:** Luciferase lysis buffer (5×)

Reagent	Amount
Glycylglycine	16.5 g
MgSO_4_ heptahydrate	18.5 g
EDTA	7.6 g
NaOH	Adjust to pH 7.8
Triton X-100	50 mL
ddH_2_O	Up to 1 L

Sterile filtrate and store at 4°C for several months.

• Media supplemented with FCS (DMEM-10): DMEM with 10% of FCS + Penicillin/Streptomycin [Store at 4°C for several weeks]

## Step-by-step method details

### Seeding TZM-bl cells

**Timing: 1 h on day 1**

Assessment of HIV-1 neutralization activity is typically performed using the pseudovirus and TZM-bl cell-based neutralization assay (short TZM-bl assay), considered a standard assay in the HIV-1 field.^2^^,^^3^ In the present protocol TZM-bl cells are kept in continuous culture in T-75 flasks and seeded in 384 well culture plates for the neutralization assay.

1. Detach cells from T-75 flask.
  a. Wash TZM-bl cells with 10 mL DPBS.
  b. Add 2 mL Trypsin-EDTA, 0.25%.
  c. Incubate cells for 5 min at 37°C, 5% CO_2_.
  d. Resuspend cells with 13 mL DMEM-10.
  e. Centrifuge cells for 10 min at 300 × *g*.
  f. Resuspend cell pellet in 10 mL fresh DMEM-10.
2. Seed cells in 384 white well, optically clear culture plates.
  a. Count cells.
  b. Seed 3.000 TZM-bl cells per well in 40 μL DMEM-10 containing 15 μg/mL Diethylaminoethyl-dextran hydrochloride (DEAE-Dextran).
3. Incubate plates overnight at 37°C, 5% CO_2_.

### ART-DEX step

**Timing: 4 h on day 2**

Antiretrovirals (ARVs) record as virus inhibition in the TZM-bl assay that is indistinguishable from antibody-based inhibition. ART-DEX has been designed to separate ARVs from plasma samples from people with HIV enabling the assessment of pure antibody-based inhibition. Most HIV-1 ARVs are highly bound to plasma proteins.^5^ ART-DEX builds on this by first releasing ARVs from plasma proteins by alkaline and acidic pH treatment followed by separation by size-exclusion filtration.

4. Treatment of plasma sample with basic pH.
  a. Dilute plasma sample 1:2.5 in phosphate buffer, pH 10.0.
  b. Incubate sample for 2 h at room temperature.

***Note:*** Exact pH of buffer solution is critical. Check pH before use of the buffer solution for ART-DEX. Troubleshooting 1.

***Note:*** We have not validated the performance of the ART-DEX step with highly viscous or fatty plasma. As all plasma samples are pre-diluted for the ART-DEX treatment, we expect that most of these plasmas can be processed without problems. We recommend however that the researchers take note of viscous/fatty plasmas at the pre-dilution step to monitor their performance in the following filter step.

5. Size-exclusion chromatography using Zeba Spin Plate, 40K MWCO.
  a. Centrifuge Zeba Spin Plate, 40K MWCO for 2 min at 700 × *g* to remove storage buffer.
  b. Add 150 μL phosphate buffer, pH 10.0 per used column to wash the column.
  c. Centrifuge Zeba Spin Plate, 40K MWCO for 2 min at 700 × *g*.
  d. Add 150 μL phosphate buffer, pH 10.0 per used column to wash the column.
  e. Centrifuge Zeba Spin Plate, 40K MWCO for 2 min at 700 × *g*.
  f. Add 150 μL phosphate buffer, pH 10.0 per used column to wash the column.
  g. Centrifuge Zeba Spin Plate, 40K MWCO for 3 min at 700 × *g*.
  h. Add 30–100 μL sample per column.
  i. Centrifuge plate for 3 min at 700 × *g*.

***Note:*** Sample loss (10–15 μL) during centrifugation may occur.

6. Treatment of plasma sample with acidic pH.
  a. Dilute eluted plasma sample 1:2 in acetate buffer, pH 3.6.
  b. Incubate sample for 1 h at room temperature.

***Note:*** Exact pH of buffer solution is critical. Check pH before use of the buffer solution for ART-DEX. Troubleshooting 2.

7. Size-exclusion chromatography using Zeba Spin Plate, 40K MWCO.
  a. Centrifuge Zeba Spin Plate, 40K MWCO for 2 min at 700 × *g* to remove storage buffer.
  b. Add 150 μL acetate buffer, pH 3.6 per used column to wash the column.
  c. Centrifuge Zeba Spin Plate, 40K MWCO for 2 min at 700 × *g*.
  d. Add 150 μL acetate buffer, pH 3.6 per used column to wash the column.
  e. Centrifuge Zeba Spin Plate, 40K MWCO for 2 min at 700 × *g*.
  f. Add 150 μL acetate buffer, pH 3.6 per used column to wash the column.
  g. Centrifuge Zeba Spin Plate, 40K MWCO for 3 min at 700 × *g*.
  h. Add 30–100 μL sample per column.
  i. Centrifuge Zeba Spin Plate, 40K MWCO for 3 min at 700 × *g*.

***Note:*** There may be sample loss (10–15 μL) during centrifugation.

8. Neutralize pH of plasma sample.
  a. Dilute eluted plasma sample 1:5 in DMEM-10 containing 25 mM HEPES.

***Note:*** We strongly recommend to directly proceed with neutralization assay after the ART-DEX step. Troubleshooting 3.

### Neutralization assay

**Timing: 2.5 h on day 2**

To determine the plasma neutralization capacity, ART-DEX-treated plasma samples are in the next steps processed as usual in the TZM-bl neutralization assay.^2^^,^^3^ In brief, samples are serially diluted, pre-incubated with pseudoviruses, then added to TZM-bl cells and incubated until the readout 48–72 h later.

9. Serially dilute ART-DEX-treated plasma samples in DMEM-10 containing 25 mM HEPES.
  a. Start dilution series with the prepared 1:5 dilution of the eluted plasma sample.
  b. Serially dilute plasma sample 1:4 in DMEM-10 containing 25 mM HEPES for 5 dilution steps.
  c. Add one well with DMEM-10 containing 25 mM HEPES only to obtain reference value for maximal infectivity of virus strain.
10. Add 25 μL pseudovirus (approx. 500 RLU/μL, diluted in DMEM-10) to the corresponding 25 μL plasma dilution. Troubleshooting 4.
11. Incubate plasma/virus mix for 1 h at 37°C, 5% CO_2_.
12. Transfer 40 μL plasma/virus mix to TZM-bl cells seeded on day 1.
13. Incubate for 48–72 h at 37°C, 5% CO_2_.

### Luciferase readout

**Timing: 30 min on day 5**

Infection of TZM-bl cells by the pseudoviruses can be quantified through the expression of the firefly luciferase reporter protein expressed upon infection.^2^^,^^3^

14. Lyse TZM-bl cells.
  a. Carefully remove media from cells.
  b. Add 25 μL Bright-Glo luciferase assay substrate (diluted 1:10 in Luciferase lysis buffer) per well.
15. Measure luciferase expression on the PerkinElmer plate reader within 30 min. Troubleshooting 5.

## Expected outcomes

ART-DEX is designed to separate ARVs from plasma samples, so that confounding effects of the ARVs on antibody neutralization measured in pseudovirus-based neutralization assays can be minimized. In this way, ART-DEX provides means to accurately measure plasma neutralization activity of ART-treated PWH.

**Figure 1:**
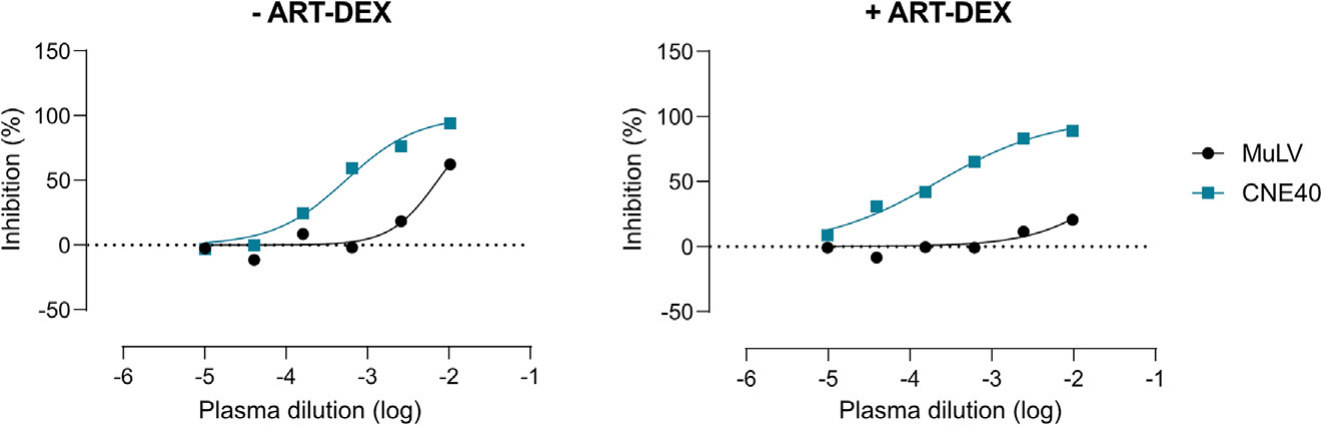
Expected outcomes after ART-DEX The data show the inhibitory activity of plasma from a person with HIV containing ARVs (Emtricitabine + Rilpivirine + Tenofovir) against MuLV pseudovirus (black) and the HIV-1 Env pseudovirus CNE40 (blue) in the standard TZM-bl based neutralization assay (left). As ARVs are present MuLV is inhibited. Analysis of the same plasma sample after the ART-DEX step in the TZM-bl assay (right) shows that ARVs are removed effectively from the plasma samples, as no MuLV inhibition is observed. The inhibition measured against CNE40 is therefore HIV-1 antibody specific.

To verify if removal of ARVs by ART-DEX was successful, neutralization against Murine Leukemia Virus (MuLV) Env pseudoviruses (or other non-HIV Env pseudovirus) should be measured. MuLV is not inhibited by plasma from PWH unless the plasma contains ARVs. Figure 1 shows an example of a successful ART-DEX treatment of a plasma sampled from a PWH on ART (Emtricitabine + Rilpivirine + Tenofovir, data from Schwarzmüller et al.,^1^; Figure 6). Without ART-DEX the plasma inhibits both MuLV pseudovirus (black) and the HIV-1 pseudovirus CNE40 (blue). With ART-DEX, ARVs are fully removed, no MuLV inhibition occurs. The inhibition measured against CNE40 is therefore solely due to HIV-1 specific antibodies in the plasma. Note in Figure 1 the wild-type pseudovirus backbone was used.

## Limitations

ART-DEX treatment is highly efficient in removing ARVs from plasma but, as we show in Schwarzmüller et al.,^1^ some residual ARV activity may be retained, depending on concentration and potency of the drug. It is thus important to verify if full ARV removal was achieved by probing the inhibitory activity of ART-DEX treated plasma against MuLV. Should residual inhibitory activity against MuLV be detected, combining ART-DEX with a multi-drug resistant pseudovirus as introduced in Schwarzmüller et al.^1^ is highly recommended.

## Troubleshooting

### Problem 1

Unstable pH for phosphate buffer solution (pH 10.0).

### Potential solution

As the pH is outside of the buffer range of phosphate buffers, the pH of the solution may be unstable. We recommend to adjust pH of the buffer solution first to pH 9.5 and adjust to pH 10.0 the next day. Re-measure pH each time before using it in the ART-DEX protocol.

### Problem 2

Unstable pH for acetate buffer solution (pH 3.6).

### Potential solution

We strongly recommend to re-measure pH each time before using the buffer in the ART-DEX protocol.

### Problem 3

We did not evaluate the impact of long-term storing pH-treated plasma samples on plasma antibodies.

### Potential solution

We strongly recommend to directly proceed with neutralization assays after ART-DEX without storing plasma samples for longer time as the pH treatment may otherwise impact plasma antibodies.

### Problem 4

Low infectivity of pseudoviruses in combination with some Envs (e.g., infectivity of pseudoviruses less than 10-fold over background signal of TZM-bl cells).

### Potential solution

Low infectivity of pseudoviruses may particularly occur when using multi-drug resistant pseudoviruses owing to their overall lower infectivity (Schwarzmüller et al.^1^). To overcome this, try to use the pseudovirus stock undiluted for the neutralization assay. We typically aim at RLU signals approx. 10-fold above background. If undiluted virus stock still does not yield enough infectivity, we recommend concentration by sucrose gradient.

### Problem 5

**Figure 2:**
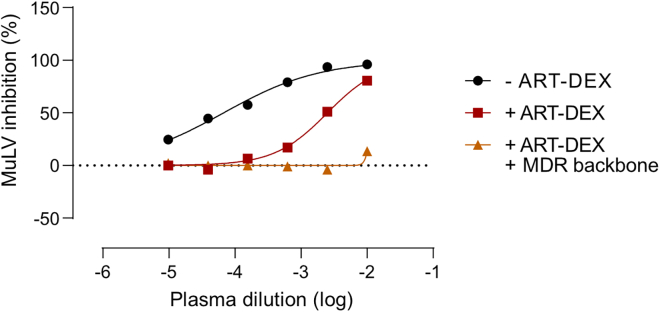
Use of multidrug-resistant backbone to overcome residual inhibitory activity of ARVs after ART-DEX The data show the inhibitory activity of plasma from a person with HIV containing ARVs (Emtricitabine + Elvitegravir + Tenofovir) against MuLV pseudovirus either in the standard TZM-bl based neutralization assay (black), after ART-DEX (red) or after ART-DEX in combination with use of the multidrug-resistant backbone HIV^pv^-MDR (orange).

Residual inhibition of wild-type MuLV pseudovirus after ART-DEX (Figure 2).

### Potential solution

If residual inhibition against wild-type MuLV pseudovirus is detected after ARV, the neutralization assay should be conducted with multidrug-resistant pseudoviruses (Schwarzmüller et al.^1^).

## Resource availability

### Lead contact

Further information and requests for resources and reagents should be directed to and will be fulfilled by the lead contact, Prof Alexandra Trkola (trkola.alexandra@virology.uzh.ch).

### Technical contact

Questions about technical specifics of the protocol should be directed to the technical contact, Magdalena Schwarzmüller (schwarzmueller.magdalena@virology.uzh.ch).

### Materials availability

The plasmid of the multidrug-resistant HIV backbone (HIV^pv^-MDR) generated in the study Schwarzmüller et al.^1^ and described in this protocol can be obtained by request to the lead author.

### Data and code availability

This study did not generate/analyze datasets or code.
